# Relationship of brain-derived neurotrophic factor, malondialdehyde, and 8-Hydroxy 2-Deoxyguanosine with post-ischemic stroke depression

**DOI:** 10.1590/1980-57642020dn14-010007

**Published:** 2020

**Authors:** Yuliarni Syafrita, Darwin Amir, Restu Susanti, I Fadhilah

**Affiliations:** 1 Neurology Department, Faculty of Medicine, Andalas University, Indonesia.; 2 Hospital, Perintis Kemerdekaan Street, Padang, West Sumatra, Indonesia.

**Keywords:** 8-OhdG, BDNF, depression, ischemic stroke, malondialdehyde, 8-OhdG, BDNF, depressão, acidente vascular cerebral isquêmico, malondialdeído

## Abstract

A few studies have shown that serum brain-derived neurotrophic factor (BDNF) level in post-stroke depression is highly correlated with memory and neuropsychiatric disturbances.

**Objective:**

This study aimed to elucidate the relationship of serum BDNF, malondialdehyde (MDA), and 8-Hydroxy 2-Deoxyguanosine (8-OhdG) levels in acute stroke cases with one-month post-stroke depression.

**Methods:**

An observational study was conducted of 72 post-ischemic stroke patients in the Neurology ward of the Dr. M. Djamil Hospital, Padang, West Sumatra, Indonesia. Acute stroke (< 48 hours) serum BDNF, MDA, and 8-OhdG levels were measured using ELISA. Based on observations using the Hamilton Depression Rating Scale conducted one month after stroke, respondents were divided into two groups: with and without depression. The mean serum level was analyzed using the *t*-test and Mann-Whitney test, while differences in basic characteristics were analyzed using the Chi-square test. Multivariate analysis was conducted to determine the most significant factor associated with post-stroke depression. The error rate was set at 5%.

**Results:**

BDNF levels in acute stroke were significantly lower in the depression group than in the non-depression group (p < 0.05). MDA and 8-OhdG levels in acute stroke were higher in the depression group (p < 0.05). BDNF level during acute stroke was negatively correlated with post-stroke depression, while, conversely, acute stroke MDA and 8-OhdG levels were positively correlated with depression.

**Conclusion:**

BDNF had a negative correlation, while MDA and 8-OhdG had a positive correlation, with depression one-month post-stroke. 8-OhdG was the most influential factor in post-stroke depression.

## RESUMO

Alguns estudos mostraram que o nível sérico de fator neurotrófico derivado do cérebro (BDNF) na depressão pós-AVC está altamente correlacionado com a memória e com os distúrbios neuropsiquiátricos.

**Objetivo:**

Este estudo teve como objetivo elucidar a relação entre os níveis séricos de BDNF, malondialdeído (MDA) e 8-hidroxi 2-desoxiganosanos (8 OhdG) em casos de AVC agudo com depressão pós-AVC de um mês.

**Métodos:**

Um estudo observacional foi realizado em 72 pacientes com AVC pós-isquêmico na enfermaria de Neurologia do Hospital Dr. M. Djamil, Padang, Sumatra Ocidental, Indonésia. Os níveis séricos de BDNF, MDA e 8-OhdG no AVC agudo (< 48 horas) foram medidos usando ELISA. Com base nas observações da Hamilton Depression Rating Scale realizada um mês após o AVC, os entrevistados foram divididos em dois grupos: com e sem depressão. O nível sérico médio foi analisado pelo teste T e Mann-Whitney, enquanto as diferenças nas características básicas foram analisadas pelo teste do qui-quadrado. A análise multivariada foi realizada para determinar o fator mais significativo associado à depressão pós-AVC. A taxa de erro foi fixada em 5%.

**Resultados:**

O nível de BDNF no AVC agudo foi significativamente menor na depressão do que no grupo sem depressão (p < 0,05). Os níveis de MDA e 8-OhdG no AVC agudo foram maiores no grupo de depressão (p < 0,05). O nível de BDNF durante o AVC agudo foi negativamente correlacionado com os casos de depressão pós-AVC, enquanto, inversamente, os níveis de MDA e 8-OhdG do AVC agudo foram positivamente correlacionados com os casos de depressão.

**Conclusão:**

O BDNF tem uma correlação negativa, enquanto o MDA e o 8-OhdG tiveram uma correlação positiva com a depressão um mês após o AVC. 8-OhdG foi o fator mais influente na depressão pós-AVC.

Post-stroke depression is a neuropsychiatric complication that frequently occurs after a stroke. The prevalence is 31% of stroke cases in < 1-year post-stroke; 25% 1-5 years post-stroke; and 23% 5 years post-stroke.^1^ This high prevalence is caused by patient dissatisfaction with the healing process, functional progress or overall outcome. Depression interferes with the healing process and is a major factor influencing stroke severity, cognitive disorder and higher general death toll.^2^^,^ 3

The pathogenesis of this depression remains unclear. Some theories, based on previous studies, attributed the depression to causes such as neurobiological issues, the patient’s behavior and habits, or social factors. Of these three factors, the neurobiological factor exhibits a different depression subtype than the others.^4^ Neurogenesis and oxidative stress are the two most hotly debated neurobiological factors that cause depression. One of the highlighted factors is neurotrophins as they regulate nerve regeneration.^5^ Neurotrophins are important signal transducer molecules in the brain, responsible for the growth and maturation of axons and neurons and for synaptic plasticity.^6^

Brain-derived neurotrophic factor (BDNF) is one of the primary neurotrophins expressed in the central and peripheral nervous system in adult mammals, especially in the hippocampus and cortex.^7^ BDNF has several functions, including the maturation and longevity of axon and dendrite growth, neurotransmitter release, and regulating long-term potentiation (LTP), and thus plays a pivotal role in regulating synaptic plasticity.^8^ It has been reported that BDNF can pass through the blood-brain barrier and its level in the brain and serum does not differ during the maturation and aging process in mice, indicating that BDNF level in serum reflects levels in the brain.^9^^.^ 10 A clinical study showed that serum BDNF level and decrease in hippocampus volume was highly correlated with memory and neuropsychiatric disorders. Further analysis also reported that low BDNF level can lead to decreased hippocampus volume and be considered the cause of spatial memory deficit and depression.^4^ BDNF injected into a depressed mouse’s brain helped alleviate the symptoms of depression.

Ischemic stroke is the most common type of stroke, reported to constitute around 85% of all stroke cases globally.^11^ This high incidence of stroke leads to a high number of cases of disability and post-stroke depression. Ischemic stroke is strongly associated with the production of reactive oxygen species (ROS) and reactive nitrogen species (RNS). ROS and RNS initiate the oxidative process in biomolecules, such as protein and DNA, creating toxic matter.^12^

It has been shown that free radicals play an important role in depression pathophysiology.^13^^-^ 15 One of the products of the oxidative reaction in brain tissue is malondialdehyde (MDA), high in unsaturated fat (polyunsaturated fatty acid/PUFA).^16^ The study by Liu stated that MDA is one of the predictive markers of post-stroke depression.^17^ The findings of Liu’s study were supported by the study by Rybka, which observed elevated MDA levels in patients diagnosed with major depression.^18^

Oxidative DNA damage is responsible for dysfunction of the blood-brain barrier and also induces the degeneration process and apoptosis. DNA damage can be predicted by elevated 8-hydroxy-2’-deoxyguanosine (8-OhdG) levels in body tissue, blood or urine. 8-OhdG is considered an important biomarker which is stable during oxidative stress to DNA.^19^^,^ 20 The study by Black found elevated 8-OhdG levels in patients with depression,^14^ in agreement with Chen’s study observing an increased 8-OhdG in the urine of subacute ischemic stroke patients diagnosed with depression.^21^ The arguments outlined above strongly suggest the connection and involvement of neurogenesis and oxidative stress with post-stroke depression cases.^6^^,^ 17 ^,^ 21 The present study was conducted to further elucidate this involvement and determine the most significant cause of post-stroke depression.

## METHODS

### Study design

This study was approved by the Ethics Committee of the Medicine Faculty, Andalas University, Padang (certificate number: 511/KEP/FK/2018). The observational study with a case control study design was conducted from January 2^nd^ to September 30^th^, 2018. The inclusion criteria were: (1) confirmed ischemic stroke patient diagnosed with brain CT scan within < 48 hours of onset; (2) age < 70 years; (3) no observed depression symptoms prior to the stroke, and post-stroke depression duration > 2 weeks; (4) no other neurodegenerative diseases, such as Alzheimer’s or Parkinson’s; and (5) willing to be included in this study. Serum levels of BDNF, MDA, and 8-OhdG were examined 48 hours post-stroke using the ELISA method. The depression assessment was based on the Hamilton Depression Rating Scale and was conducted one month after the stroke at the Neurology Polyclinic of the Dr. M. Djamil Hospital, Padang, West Sumatra, Indonesia. Based on the depression scale assessment, the patients were divided into two groups (36 people in each): post-stroke with depression and without depression.

### BDNF, MDA, and 8-OhdG examination

Total serum BDNF level was measured with an ELISA kit (sandwich ELISA method). The ELISA microplate was coated with anti-BDNF antibody and the BDNF signal was detected using biotinylated specific antibody and Avidin-Horseradish Peroxidase (HRP). MDA and 8-OhdG levels were measured with the competitive ELISA strategy. The ELISA microplate was coated with the respective target (MDA or 8-OhdG). During the reaction, MDA/8-OhdG in the standard plate competed with the serum MDA/8-OhdG for the specialized Biotinylated Detection Ab for MDA/8-OhdG. Subsequently, the measurement procedure was based on the manufacturer’s instruction. The color change at the end of the reaction was measured using spectrophotometry at a wavelength of 450 + 2 nm.

### Statistical analysis

The basic differences between the two groups were assessed using Chi-Square and Mann-Whitney tests. Mean differences for each variable (BDNF, MDA, and 8-OhdG) in the two groups were tested using the *t*-test for normally distributed data and the Mann-Whitney test for non-normally distributed data. Multiple analysis with linear regression was conducted to determine the highest correlation of variables (marital status, education level, side of lesion, MDA, BDNF and 8-OhdG) with post-stroke depression. Statistical tests were considered significant for p-value < 0.05.

## RESULTS

Based on the Chi-square test, the basic characteristics of the two groups were statistically similar (p > 0.05) (Table 1).

**Table 1:** Basic characteristics of the two groups.

	Post-Ischemic Stroke		
	with depression	without depression	p
Age	59.67 ± 9.7	59.64 ± 11.2	0.85*
Gender M/F	18/18	19/17	0.814*
Education level: low/high	18/18	12/24	0.151*
Marriage status: married/single	32/4	31/5	0.722*
Left/Right lesion	11/25	19/17	0.056*

* Chi-square test.

Mean serum BDNF level in depressed patients was significantly lower (p < 0.05) than in patients without symptoms of depression. Serum MDA and 8-OhdG levels were significantly higher (p < 0.05) in the post-stroke patients with symptoms of depression than in those who did not have depression (Table 2).

**Table 2:** Comparison of BDNF, MDA and 8-OhdG levels in the two groups.

Variable	Post-Ischemic Stroke		
	with depression	without depression	P
BDNF (pg/ml)	6442.50 ± 1747.48	7522.33 ± 1638.45	0.009*
MDA (ng/ml)	110.06 ± 33.27	99.98 ± 54.76	0.024**
8-OhdG (ng/ml)	4.39 ± 2.19	3.08 ± 0.73	< 0.001**

* t- Test;

** Mann-Whitney test.

The correlation between BDNF level and post-stroke depression was negative and statistically significant (p < 0.05). Conversely, MDA and 8-OhdG had a positive correlation with post-stroke depression, which was also statistically significant (p < 0.05) (Table 3).

**Table 3:** Serum BDNF, MDA, and 8-OhdG level correlations with post-stroke depression.

Variable	Post-stroke depression	
	r	p
BDNF	-0.308	0.009
MDA	0.268	0.023
8-OhdG	0.432	< 0.001

As shown in Table 4, the variable with the strongest correlation with post-stroke depression was 8-OhdG, followed by BDNF, and MDA.

**Table 4:** Multiple analysis using linear regression of serum level with post-stroke depression.

Variable	Coefficient	Correlation coefficient	p
BDNF	-5.818	-0.204	0.074
8-OhdG	0.087	0.302	0.009
Right-sided lesion	0.193	0.191	0.081
Constant			< 0.001

## DISCUSSION

The statistical analysis of the basic characteristics of the two groups returned a p-value higher than 0.05 (p > 0.05), indicating the groups were similar. The serum BDNF level of the depressed post-stroke patients was significantly lower than the post-stroke patients without depression. Li’s 2016 study of 216 ischemic stroke patients showed that a low BDNF serum level on hospital admission could serve as an independent predictor of post-stroke depression at three-month follow-up.^22^ A meta-analysis also showed a lower BDNF level in post-stroke depression patients.^23^

The survival and development of neuron cells in the central nervous system are regulated by numerous extracellular factors, among which are neurotrophins. One of the neurotrophins in the BDNF, which facilitates the growth and sustainability of nerve cells, modulates synapse response and is responsible for synapse plasticity.^24^ A recent study showed that failure of synapse neuroplasticity (neurogenesis, axon branching, dendritogenesis, and synaptogenesis) in particular regions of the brain, especially the hippocampus, is the main pathophysiological factor in depression. This failure is closely related with changes in neurotrophins like BDNF, responsible for synapse plasticity.^25^

Decreased mRNA BDNF expression in the hippocampus and prefrontal cortex (PFC) was observed in a depressed animal model. In chronic ischemic cases, the sensitivity and density of the glucocorticoid receptor decreases as a result of the continuous increase in blood cortisol. The high level of cortisol causes atrophy and decreases synaptic density in the hippocampus, effectively decreasing the neurotransmitter release regulated by BDNF, eventually leading to disorders in learning, memory, and other cognitive functions, besides causing depression.^26^

Lipid peroxidation can be measured using several markers, including MDA, which is more stable relative to other biomarkers. MDA is the main product of lipid peroxidation, and increases in major depression patients.^27^ Several studies have reported that free radicals are also involved in depression pathophysiology.^17^^,^ 28 ^,^ 29

This study observed higher MDA levels in the depressed post-stroke group compared to the group without depression (p = 0.024). This is due to the vulnerability of brain tissue to oxidative stressors, given its high oxygen consumption and the high PUFA content of brain tissue. Both these factors increase toxic peroxidation lipid production, which can kill many types of cells.^12^ Liu observed a correlation of high MDA levels in acute (< 24 hours) stroke with 1-month post-stroke depression, especially in minor stroke cases.^17^

Besides high MDA levels, the present study also observed higher serum 8-OhdG levels in post-stroke patients with depression (p < 0.001). 8-OhdG is one of the markers of nuclear and mitochondrial DNA damage due to oxidative stress. This type of DNA damage is known to cause dysfunction of the blood-brain barrier and can eventually lead to cell degeneration and apoptosis.^30^

The role of 8-OhdG in the pathophysiology of post-stroke depression is as yet unclear, but several theories propose that it could be caused by an imbalance between pro-oxidants and antioxidants. The ROS increase in the brain during hypoxic conditions causes excessive oxidation to lipids and proteins and damages the DNA of nerve cells. Prolonged oxidative stress can lead to dysregulation in the hypothalamus-pituitary-adrenal axis, one of the main stress regulating systems.^14^ Lifestyle, age, smoking, and dysregulation in the hypothalamus-pituitary-adrenal axis have all been reported in depression cases. A combination of these factors can be considered the main mechanism of post-stroke depression.^12^^,^ 30 ^,^ 31 Liu’s study also found that elevated 8-OhdG in the acute phase was correlated with one-month post-stroke depression.^29^ These arguments imply that peripheral 8-OhdG levels can serve as an indicator of the oxidative process in the brain.^30^

In this study, it was found that MDA serum level was significantly higher in the post-stroke depression group than in the non-depression group (p=0.024). Brain is a vulnerable tissue that can be easily damaged by oxidative stress, because brain tissue metabolism needs a high amount of oxygen and is rich in polyunsaturated fatty acid/(PUFA). This damage causes lipid peroxidation and leads to the death of various types of cells. There are several markers used to measure lipid peroxidation process, of which malondialdehyde is one of the more stable. Malondialdehyde is the main product of lipid peroxidation and its concentration increases in patients with major depression. We also found significantly higher 8-OhdG serum levels in the post-stroke depression group compared to the non-depression group (p= < 0.001). The role of 8-OhdG in post-stroke depression is still a question of debate. Some of the recent theories suggest that 8-OhdG´s role in post-stroke depression correlates with pro-oxidant-antioxidant imbalance. Hypoxemia increases ROS level in the brain. The increasing level of ROS causes lipid peroxidation, protein peroxidation and neuronal DNA damage. Overall, this pathophysiology can be considered the main mechanism inducing post-stroke depression. 8-hydroxy 2-deoxy guadenosine is one of the products of DNA and mitochondrial damage product, rendering it an oxidative stress process marker. Multiple analysis of the three main variables showed that 8-OhdG serum level had the highest correlation with post-stroke depression , followed by BDNF and MDA. This result suggests that DNA damage, due to the oxidative process in the acute phase of stroke, is more sensitive as a marker of the severity of the damage. A previous study also reported that 8-OhdG levels in the acute phase of stroke infarction are an important marker of the severity of brain damage due to oxidative processes.^30^

This study has several limitations in that it did not analyze other factors that may contribute to depression, such as lesion size, inflammation, neurotransmitter factors or comorbidities.

In conclusion, this study observed that BDNF serum level has a negative correlation, while serum MDA and 8-OhdG levels have a positive correlation with depression one-month post-stroke. 8-OhdG is the factor that most strongly correlates with post-stroke depression.^32^
